# Two populations of less-virulent *Helicobacter pylori* genotypes in Bangladesh

**DOI:** 10.1371/journal.pone.0182947

**Published:** 2017-08-10

**Authors:** Hafeza Aftab, Muhammad Miftahussurur, Phawinee Subsomwong, Faruque Ahmed, A. K. Azad Khan, Takashi Matsumoto, Rumiko Suzuki, Yoshio Yamaoka

**Affiliations:** 1 Department of Environmental and Preventive Medicine, Oita University Faculty of Medicine, Yufu, Japan; 2 Department of Gastroenterology, Dhaka Medical College and Hospital, Dhaka, Bangladesh; 3 Gastroentero-Hepatology Division, Department of Internal Medicine, Faculty of Medicine-Dr. Soetomo Teaching Hospital-Institute of Tropical Disease, Airlangga University, Surabaya, Indonesia; 4 Department of Medicine, Gastroenterology and Hepatology Section, Baylor College of Medicine, Houston, Texas, United States of America; 5 Department of Gastroenterology, Bangladesh Institute of Research and Rehabilitation in Diabetes, Endocrine and Metabolic Disorders, Dhaka, Bangladesh; National Cancer Center, JAPAN

## Abstract

Bangladesh has a population with a low gastric cancer risk but high prevalence of *Helicobacter pylori* infection. Several studies have examined virulence genes in *H*. *pylori* from Bangladesh. We analyzed *cagA* and *vacA* subtypes and their association with severe histology phenotypes, and analyzed population types among Bangladeshi strains. We included patients who underwent endoscopy in Dhaka. Sequences of virulence genes and seven housekeeping genes were obtained by next generation sequencing and confirmed by Sanger sequencing. We isolated 56 *H*. *pylori* strains from 133 patients, of which 73.2% carried *cagA*, and all were considered Western-type. Patients infected with *cagA*-positive strains had more severe histological scores than patients infected with *cagA*-negative strains. Among *vacA* s1 and m1 genotypes, the s1a (97.8%, 43/44) and m1c (28/30, 93.3%) genotypes were predominant. All strains containing s1 and m1 (30/56, 53.6%) also had i1, d1, and c1. In contrast, all strains containing the less-virulent genotypes s2 and m2 (12/56, 21.4%) also possessed i2, d2, and c2. Multivariate analysis indicated that subjects infected with *vacA* m1-genotype strains only had a significantly higher risk of antrum atrophy than patients infected with m2-genotype strains. Of the two main *H*. *pylori* populations in this study, hpAsia2 strains were associated with higher activity and inflammation in the antrum compared to hpEurope strains; however, only *vacA* s1m1i1d1c1 strains, independent of population type, were significantly associated with inflammation in the antrum, unlike the s2m2i2d2c2 genotype. In conclusion, Bangladeshi strains were divided into two main populations of different genotypes. The low incidence of gastric cancer in Bangladesh might be attributable to the high proportion of less-virulent genotypes, which may be a better predictor of gastric cancer risk than the ancestral origin of the *H*. *pylori* strains. Finally, the *vacA* m region may be a better virulence marker than other regions.

## Introduction

*Helicobacter pylori*, a gram-negative bacterium responsible for several gastroduodenal diseases, produces a number of virulence factors that are essential for colonization of the stomach and survival in the hostile gastric environment [1]. A well-known virulence factor is *cagA*, which encodes CagA, a 120–145-kDa immunodominant protein [2]. CagA activates a number of signal transduction pathways that are involved in binding and perturbing the function of epithelial junctions, resulting in aberrations in tight junction function, cell polarity, and cellular differentiation [3]. *H*. *pylori* strains can be categorized as *cagA*-positive or -negative. CagA expression in *cagA*-positive strains has been associated with inflammation and an increased risk for more severe clinical outcomes when compared to *cagA*-negative strains in *H*. *pylori*-infected patients [1]. *cagA*-positive strains that have EPIYA motifs, which are tyrosine-phosphorylated by Src and Abl family kinases, impair a variety of intracellular signaling systems after they infect gastric epithelial cells [4]. An increased risk of gastric cancer is observed in individuals infected with strains possessing *cagA* with an EPIYA-D segment (an East Asian-type *cagA*-positive strain) than with strains possessing an EPIYA-C segment (a Western-type *cagA*-positive strain) [1].

The CagA multimerization (CM) sequence is located within the EPIYA-C segment (FPLKRHDKVDDLSKVG) and, downstream of the EPIYA-D segment (FPLRRSAAVNDLSKVG). The type and number of CM motifs may affect the potential for multimerization of individual CagA proteins in host cells, which may, in turn, affect the ability of CagA to disturb host cell function via Src homology region 2-containing protein tyrosine phosphatase 2 (SHP2) deregulation [5]. An amino acid sequence alignment of the pre-EPIYA region of *cagA*, located approximately 300-bp upstream of the first EPIYA motif, revealed that a 39-bp deletion was present in most strains isolated from East Asia. This deletion was absent in most strains from Western countries, which were subsequently denoted as the no-deletion type of *H*. *pylori* [6, 7].

Virulent strains of *H*. *pylori* also produce vacuolating cytotoxin A (VacA), which enters host cells via endocytosis and induces multiple cellular activities, including membrane channel formation, cytochrome *c* release from mitochondria leading to apoptosis, and binding to cell membrane receptors that initiates a proinflammatory response [8]. The gene encoding *vacA* displays allelic diversity, including diversity in the signal (s) regions s1 and s2 and in the middle (m) regions m1 and m2. Based on *in vitro* experiments, s1m1 strains are the most cytotoxic, as they consistently induce cell vacuolation. s1m2 strains do not consistently induce cell vacuolation, and s2m2 strains show no cytotoxic activity [1]. The s1 and m1 types can be further subdivided into s1a, s1b, and s1c, and m1a, m1b, and m1c, respectively [1].

The intermediate (i) region is a third disease-related region of *vacA*. It is located between the s region and the m region and is divided in two subtypes: i1 and i2. It has been suggested that the i region may be a better predictor of disease severity than either the s or the m region [9, 10]. The deletion (d) region, located between the i and m regions, remains poorly studied. The 69–81-bp deletion (d2) type was reported to be less virulent than the no-deletion (d1) type [11]. One study reported that the frequency of the *vacA* d1 genotype was 43.4% and that its frequency was significantly higher in *H*. *pylori* isolates from patients with peptic ulcer disease (71.4%) than in isolates from patients with gastritis (27.4%) [12]. Recently, a novel polymorphic site in the 3′ end of *vacA*, denoted as c1 and c2 (with c1 being more virulent than c2), was reported to be a strong predictor of gastric cancer in male patients [13].

Multi-locus sequence typing (MLST) using seven housekeeping genes identified seven population types: hpEurope, hpEastAsia, hpAfrica1, hpAfrica2, hpAsia2, hpNEAfrica, and hpSahul; these strains reflect geography and human migration out of Africa [14–16]. Interestingly, in addition to virulence factors, population types were reported to be closely associated with the incidence of gastric cancer and partly explained the “Asian enigma,” the phrase that refers to an area with a low incidence of gastric cancer despite a high prevalence of *H*. *pylori* infection [17]. A previous study suggested that a European-origin strain (hpEurope) was associated with premalignant histological lesions, while infection with African strains (hpAfrica1) was not [18, 19]; however, there have been no previous studies focusing on the relationship between the phylogeographic origin of *H*. *pylori* and gastric cancer risk in Asia, particularly for hpAsia2 [17].

Bangladesh is a South Asian country with a population of more than 160 million, making it the world's eighth most populous country. The prevalence of *H*. *pylori* infection is reportedly high (60.2%), which is similar to that of other developing countries (53.8% among those aged 12–19 years) [20]. However, the age-standardized incidence rate (ASR) of gastric cancer in Bangladesh is low for Asian countries (5.4/100,000; available from the International Agency for Research on Cancer; GLOBOCAN2012, http://globocan.iarc.fr/). Several studies have characterized Bangladeshi strains based on their *cagA* and *vacA* status [21–23], but there have been few studies that have analyzed the association of *cagA* or *vacA* subtypes with histological scores of disease severity. Therefore, in this study, we analyzed *cagA* and *vacA* subtypes in Bangladeshi strains and their association with severe histological scores. Moreover, we analyzed Bangladeshi strains using seven housekeeping genes and identified two predominant populations. The results of this study might explain the low gastric cancer prevalence in Bangladesh.

## Materials and methods

### Patients

This study enrolled patients who underwent endoscopy at Dhaka Medical College in November 2014. Exclusion criteria were the following: a history of partial gastric resection; previous eradication therapy for *H*. *pylori*; or treatment with bismuth-containing compounds, H2-receptor blockers, or proton pump inhibitors (PPI) in the previous four weeks. Experienced endoscopists collected gastric biopsy specimens during each endoscopy session, including two samples (for culture and histology) from the lesser curvature of the antrum approximately 3 cm from the pyloric ring and one sample from the greater curvature of the corpus (for histology). Peptic ulcer diseases, including gastric and duodenal ulcers, were diagnosed by endoscopic observation, while chronic gastritis was diagnosed by histologic examination. Biopsy specimens for bacterial culture were immediately placed at −20°C and stored at −80°C within a day of collection for later use. Written informed consent was obtained from all participants, and the protocol was approved by the Ethics Committee of the Bangladesh Medical Research Council (BMRC), Dhaka, Bangladesh, and the Oita University Faculty of Medicine, Japan.

### Histology and immunohistochemistry

One antrum and one corporal specimen from each patient was histologically examined. Biopsy materials were fixed in 10% buffered formalin and embedded in paraffin. Serial sections were stained with hematoxylin and eosin, as well as with May and Giemsa stains. The degree of inflammation, neutrophil activity, atrophy, intestinal metaplasia, and bacterial density were classified into four grades, in accordance with the updated Sydney system: 0, ‘normal’; 1, ‘mild’; 2, ‘moderate’; and 3, ‘marked’ [24]. Samples with grades of 1 or higher of atrophy were considered atrophy-positive. In addition, the gastritis stage was assessed based on the topographic location (antrum or corpus) of atrophy according to the Operative Link on Gastritis Assessment (OLGA) system [25].

Immunohistochemistry was performed as previously described [26]. Briefly, after antigen retrieval and inactivation of endogenous peroxidase activity, tissue sections were incubated overnight at 4°C with anti-*H*. *pylori* antibody (Dako, Glostrup, Denmark), anti-CagA antibody (b-300, Santa Cruz Biotechnology, Dallas, TX, USA), or anti-East Asian-type CagA-specific antibody (α-EAS Ab), which was immunoreactive only with the East Asian-type CagA strains and not with Western-type CagA strains [26], diluted 1:2000 (Dako). After washing, the sections were incubated with biotinylated goat anti-rabbit or anti-rat IgG (Nichirei Co., Tokyo, Japan), which was followed by an incubation with a solution of avidin-conjugated horseradish peroxidase (Vectastain Elite ABC Kit; Vector Laboratories Inc., Burlingame, CA, USA). Peroxidase activity was detected using an H_2_O_2_/diaminobenzidine substrate solution.

### *H*. *pylori* isolation and genotyping

For *H*. *pylori* culture, antral biopsy specimens were homogenized and inoculated onto antibiotics selection plates and were subsequently subcultured on Mueller Hinton II Agar medium (Becton Dickinson, Franklin Lakes, NJ, USA) supplemented with 10% horse blood without antibiotics (Nippon Biotest Laboratories Inc., Tokyo, Japan). The plates were incubated for up to 10 days at 37°C under microaerophilic conditions (10% O_2_, 5% CO_2_, and 85% N_2_). *H*. *pylori* isolates were identified based on colony morphology; Gram staining results; and oxidase, catalase, and urease reactions. Isolated strains were stored at −80°C in Brucella Broth (Difco, Franklin Lakes, NJ, USA) containing 10% dimethyl sulfoxide and 10% horse serum (Nippon Biotest Laboratories Inc., Tokyo, Japan).

Genomic DNA was extracted from confluent plate cultures expanded from a single colony of *H*. *pylori* using a commercially available kit (Qiagen, Valencia, CA, USA) according to the manufacturer’s directions. DNA samples were subjected to next generation sequencing (NGS) (Illumina, Inc., San Diego, CA). MiSeq output was used to create contig sequences using CLC Genomics Workbench 7.0.4 (CLC Bio-Qiagen, Aarhus, Denmark). Genomics Workbench was also used to analyze seven housekeeping genes (*atpA*, *efp*, *mutY*, *ppa*, *trpC*, *ureI*, and *yphC*), full-length *cagA*, and full-length *vacA*, with sequences confirmed by polymerase chain reaction (PCR) amplification and direct sequencing, as described previously [27]. DNA sequencing was performed using a Big Dye Terminator v3.1 Cycle Sequencing Kit on an AB 3130 Genetic Analyzer (Applied Biosystems, Foster City, CA, USA) according to the manufacturer’s instructions.

### Analysis of the *H*. *pylori* population structure

Seven multi-locus sequence types of genes in the genomic DNA of Bangladeshi strains and 430 representative MLST sequences representing various populations were downloaded from the PubMLST database (http://pubmlst.org/). A neighbor-joining tree (Kimura’s two-parameter model) was constructed using the dataset. Next, using similar strains, we analyzed the bacterial population structure using STRUCTURE (v.2.3.2) software [28]. Markov Chain Monte Carlo (MCMC) simulations were run in STRUCTURE using a non-admixture model with a burn-in of 20,000, followed by 30,000 iterations for each run.

### Data analysis

Discrete variables were tested using a chi-square test; continuous variables were tested using Mann-Whitney *U* tests. A multivariate logistic regression model was used to calculate the odds ratios (OR) of various clinical outcomes, with age, sex, and *H*. *pylori* genotypes as predictors. All determinants with P values less than 0.10 were included in the full logistic regression model, and the model was reduced by excluding variables with P values greater than 0.10. OR and 95% confidence intervals (CI) were used to estimate risk. A P value less than or equal to 0.05 was considered statistically significant. SPSS statistical software package version 18.0 (SPSS, Inc., Chicago, IL, USA) was used for all statistical analyses.

## Results

### Prevalence of *H*. *pylori*

From 133 consecutive patients (61 males and 72 females; age range, 18 to 65 years; mean age, 35.2 ± 11.8 years), a total of 56 *H*. *pylori* strains were isolated: 38 from patients living in Dhaka city and 18 from patients living in the village outside of Dhaka city. All of the patients were ethnically Bengali. Isolates were obtained from 26 male (age range, 18 to 56 years; mean age, 34.2 ± 11.6 years) and 30 female patients (age range, 19 to 65 years; mean age 36.1 ± 12.1 years). Of these patients, 53 had chronic gastritis, and three had peptic ulcer diseases. No association between age, sex, and diagnosis was found (P >0.05).

### Virulence genes

In total, 41 of 56 strains possessed *cagA* (73.2%). *cagA* in two strains was not detected by either NGS or PCR; however, these strains contained partial *cag* pathogenicity island (PAI) genes, as confirmed by PCR of the *cag* PAI empty site. Therefore, we considered these strains to be “*cagA* undetermined”. Thirty-nine of 41 strains positive for *cagA* were confirmed to be immunoreactive with anti-CagA antibody by immunohistochemistry.

Based on NGS results, which were confirmed by PCR-based sequence analyses, the *cagA* genotypes included 27 ABC, 8 ABCC, 2 ABCCC, and 1 ABBC types (Table 1). The remaining three strains were AB type. All of the strains were considered Western-type *cagA*. The AB type was regarded as Western-type *cagA* based on the similarities of its B segment sequences with those of Western-type *cagA* sequences [29]. In agreement with the sequencing results, all *cagA*-positive strains showed no immunoreactivity to the α-EAS Ab, which is specific for East Asian-type *cagA*.

**Table 1:** *Helicobacter pylori* virulence factors and clinical outcome.

Description of genotype	Total		Gastritis		Peptic ulcer disease	
	No.	%	No.	%	No.	%
Total studied	56		53		3	
Mean age (yr)	35.2 ± 11.8		35.4 ± 11.9		31.7 ± 10.4	
Sex						
Female	30	53.6%	28	93.3%	2	6.7%
Male	26	46.4%	25	96.2%	1	3.8%
*cagA*						
*cagA*-positive	41	73.2%	39	95.1%	2	4.9%
AB	3	7.3%	3	100.0%	0	0.0%
ABC	27	65.9%	26	96.3%	1	3.7%
ABBC	1	2.4%	1	100.0%	0	0.0%
ABCC	8	19.5%	7	87.5%	1	12.5%
ABCCC	2	4.9%	2	100.0%	0	0.0%
*cagA*-negative	13	23.2%	12	92.3%	1	7.7%
*cagA* undetermined	2	3.6%	2	100.0%	0	0.0%
Pre-EPIYA type (no deletion)	41	100.0%	39	95.1%	2	4.9%
*vacA*						
s1	44	78.6%	41	93.2%	3	6.8%
s2	12	21.4%	12	100.0%	0	0.0%
m1	30	53.6%	30	100.0%	0	0.0%
m2	26	46.4%	23	88.5%	3	11.5%
i1	38	67.9%	36	94.7%	2	5.3%
i2	18	32.1%	17	94.4%	1	5.6%
d1	37	66.1%	35	94.6%	2	5.4%
d2	19	33.9%	18	94.7%	1	5.3%
c1	30	53.6%	30	100.0%	0	0.0%
c2	26	46.4%	23	88.5%	3	11.5%
*vacA* s1m1i1d1c1	30	53.6%	30	100.0%	0	0.0%
s1m2i1d1c2	7	12.5%	5	71.4%	2	28.6%
s1m2i1d2c2	1	1.8%	1	100.0%	0	0.0%
s1m2i2d2c2	6	10.7%	5	83.3%	1	16.7%
s2m2i2d2c2	12	21.4%	12	100.0%	0	0.0%
*cagA*-positive/*vacA* s1m1i1d1c1	28	68.3%	28	100.0%	0	0.0%
s1m2i1d1c2	7	17.1%	5	71.4%	2	28.6%
s1m2i1d2c2	1	2.4%	1	100.0%	0	0.0%
s1m2i2d2c2	3	7.3%	3	100.0%	0	0.0%
s2m2i2d2c2	2	4.9%	2	100.0%	0	0.0%
*cagA* undetermined/*vacA* s1m1i1d1c1	2	100.0%	2	100.0%	0	0.0%
*cagA*-negative*/vacA* s1m2i2d2c2	3	23.1%	2	66.7%	1	33.3%
s2m2i2d2c2	10	76.9%	10	100.0%	0	0.0%

EPIYA motifs were also evaluated (Table 2). In total, 134 EPIYA motifs were identified in the 41 *cagA*-positive strains. In agreement with our previous studies [27, 29], we found two types of motifs: EPIYA (130/134, 97.0%) and EPIYT (4/134, 3.0%). The EPIYA-B contained two types of motifs that included five amino acids (EPIYA and EPIYT), and EPIYT was found only in this motif. After excluding three strains of the AB type, we analyzed 38 CM motifs in Western-type *cagA* strains in the EPIYA-B segment and in the first distal repeat of the EPIYA-C segment (Table 3). The majority of the CM motifs in Western-type *cagA* strains were somewhat different from the typical CM motifs (FPLKRHDKVDDLSKVG) observed in strains circulating in Western countries; for examples the first motifs were divergent (20/38, 52.6%), including FPLK**K**HDKVDDLSKVG or **Y**PLKRHDKVDDLSKVG, but the second motif was not (30/38, 78.9% typical sequences). Most of the first motif sequences were identical to those in the second motif (20/38, 52.6%); for example, both motifs were FPLKRHDKVDDLSKVG. Sequence analyses of the 300-bp region upstream of the first EPIYA motif, called the pre-EPIYA, revealed that no strains contained a deletion that is typically observed in strains from Western countries.

**Table 2:** Frequencies of EPIYA motifs in Bangladeshi strains.

All motifs	No.	A motif	No.	B motif	No.	C motif	No.
EPIYA	130	EPIYA	41	EPIYA	38	EPIYA	51
EPIYT	4			EPIYT	4		
Total	134		41		42		51

**Table 3:** CagA multimerization (CM) motif in Western-type *cagA H*. *pylori* strains from Bangladesh.

Peptide sequences			
1^st^ motif	No.	2^nd^ motif	No.
FPLKRHDKVDDLSKVG	18	FPLKRHDKVDDLSKVG	30
FPLKK*HDKVDDLSKVG	17	FPLKK*HDKVDDLSKVG	4
Y*PLKRHDKVDDLSKVG	3	Others	4
Total	38	Total	38

*Bold letters are used to highlight the different between typical and atypical CM motifs.

**Fig 1:**
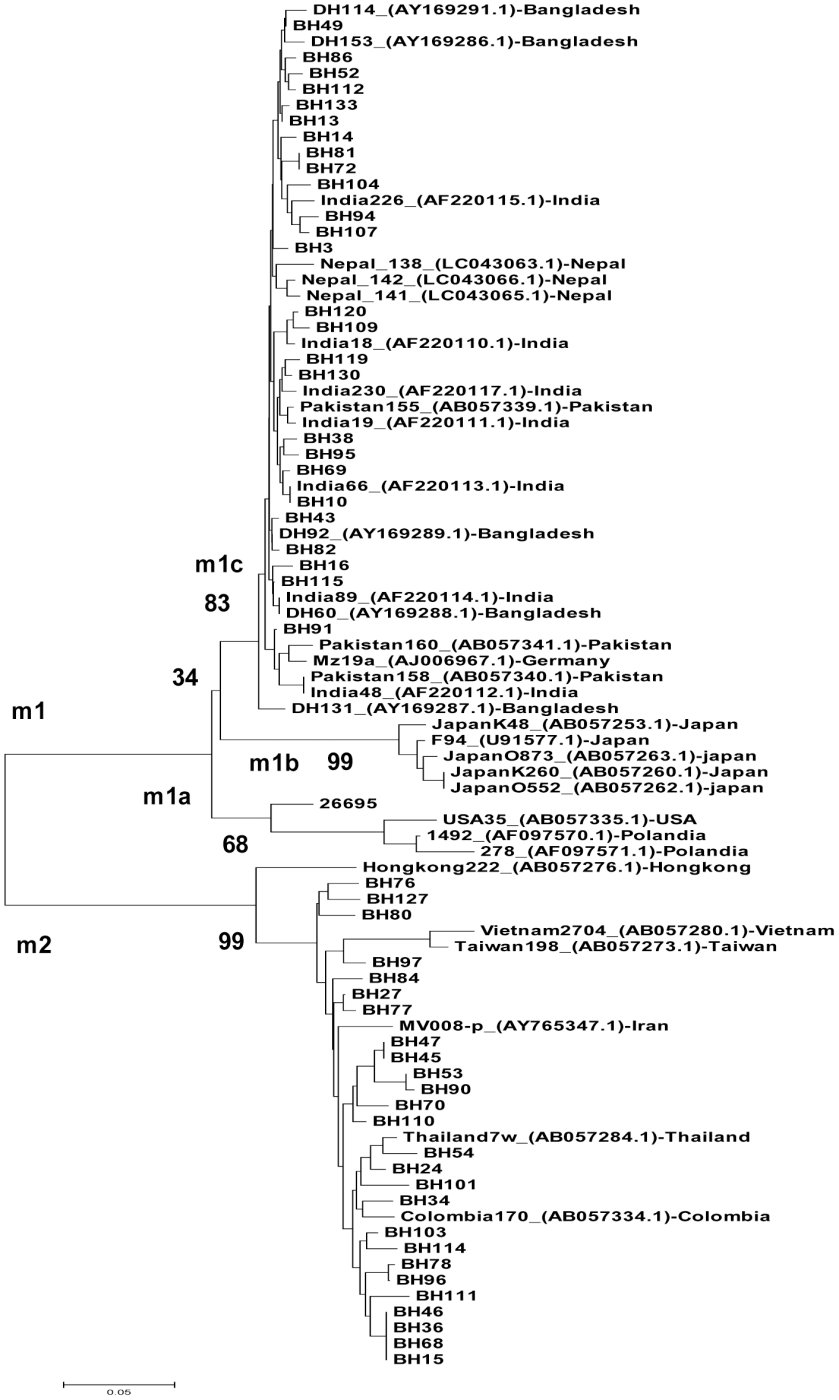
Phylogenetic tree of *Helicobacter pylori* nucleotide sequences, including the *vacA* m region. Genetic distances were estimated using the six-parameter method, and phylogenetic trees were constructed using the neighbor-joining method. Reference strains are shown with strain names and GenBank accession numbers. Bootstrap values are shown along each main branch of the tree. The lengths of the horizontal bars indicate the number of nucleotide substitutions per site.

The predominant *vacA* s type was s1 (44/56, 78.6%), with 97.8% (43/44) of these being genotype s1a (Table 1), which is typical of South Asia strains. The remaining strain (BH15) was not classified in any subtype by the *vacA* signal region. The *vacA* s2 genotype accounted for 21.4% (12/56) of the isolates. The prevalence of the *vacA* m1 genotype was 53.6% (30/56), while the *vacA* m2 genotype accounted for 46.4% (26/56) of all strains. Results of a sequence analysis of the 0.7-kb m region of *vacA* were consistent with PCR results and indicated a clear distinction between the m1 and m2 sequences (Fig 1). The genotype m1c, a typical South Asian genotype, was predominant (28/30, 93.3%), with no strains of genotype m1a or m1b detected. The remaining two strains (BH73 and BH63) were not classified as any subtype, based on the *vacA* m region. Detailed data on the *vacA* i, c, and d regions are shown in Table 1.

When examining combinations of the *vacA* genotypes, it was found that all strains that contained s1 and m1 also possessed i1, d1, and c1 (30/56, 53.6%). In contrast, all strains that contained the less-virulent genotypes s2 and m2 possessed i2, d2, and c2 (12/56, 21.4%), and all strains that possessed s1m2i2 also contained d2 and c2 (6/56, 10.7%) (Table 1). An examination of strains that had both *cagA* and *vacA* showed that all *cagA*-negative strains possessed m2i2d2c2, while m1i1d1c1 was predominant (28/41, 68.3%) in *cagA*-positive strains.

### Histology

**Fig 2:**
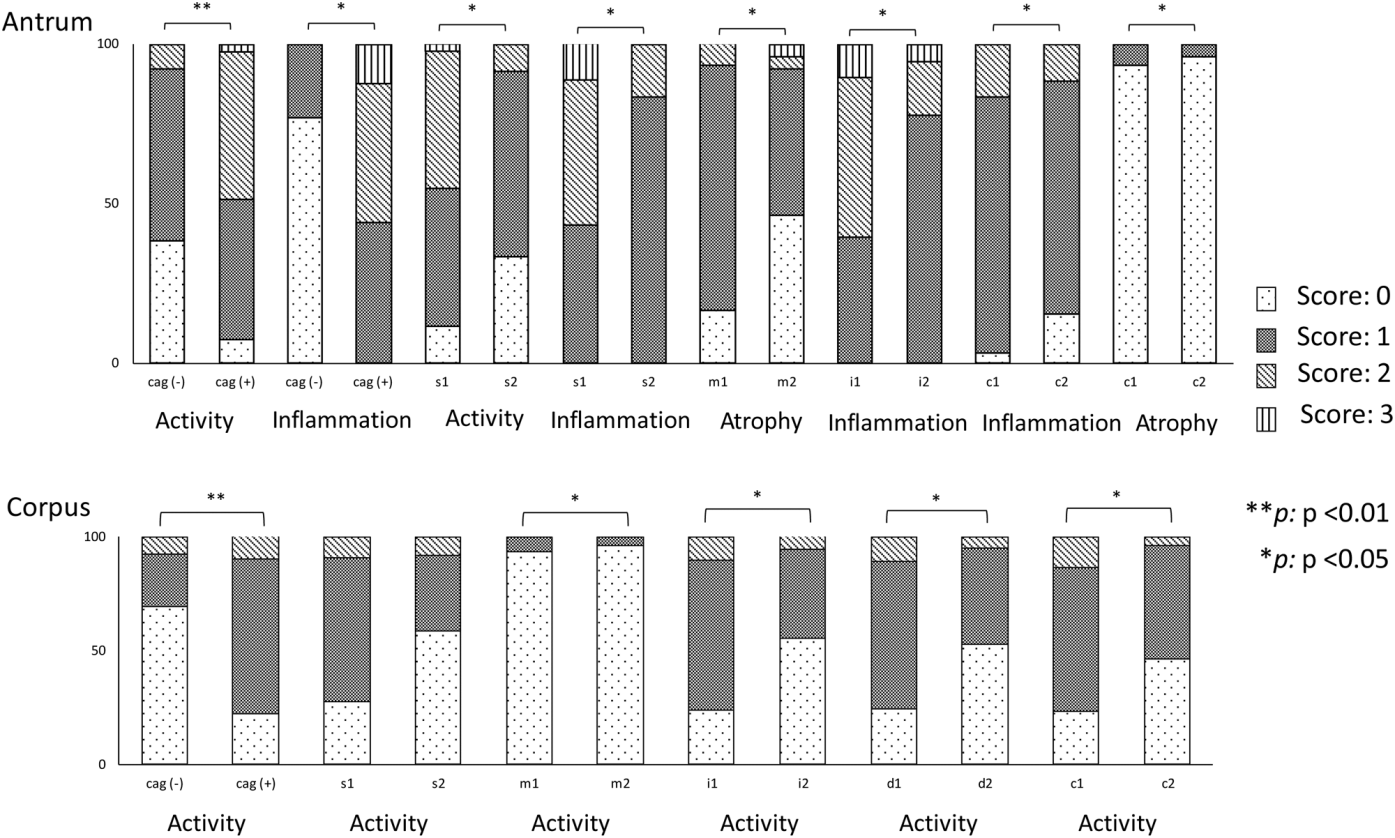
Association between histological findings and genotypes in *H*. *pylori* strains from Bangladesh. The X-axis describes the degree of histological severity, which was classified into four grades based on the updated Sydney system (scores from 0 to 3), and the Y-axis indicates the genotypes of *cagA* and *vacA*. *cagA*-positive strains induced more severe histological damage in the antrum and corpus. Similar results were also found for *vacA* genotypes, including s1 vs. s2 only in the antrum, and m1 vs. m2, i1 vs. i2, c1 vs. c2, and d1 vs. d2 in both the antrum and corpus.

Of the 56 patients infected with *H*. *pylori*, only 3 (5.4%) and 1 (1.8%) patients suffered atrophic gastritis and intestinal metaplasia, respectively. Histological analysis (Fig 2) showed that activity in the antrum and corpus was significantly greater in subjects infected with *cagA*-positive strains than in those infected with *cagA*-negative strains (mean [median]; 1.23 [1] vs. 0.69 [1], P = 0.001 and 0.75 [1] vs. 0.38 [0], P = 0.007, respectively). In addition, inflammation in the antrum was also significantly greater in subjects infected with *cagA*-positive strains than in those infected with *cagA*-negative strains (1.57 [1] vs. 1.23 [1], P = 0.03). Compared to subjects infected with *cagA*-negative strains, those infected with *cagA*-positive strains had a significantly higher risk of antrum and corpus activity after adjusting for age and sex (OR = 7.56, 95% CI = 1.46 to 39.2 and OR = 9.15, 95% CI = 2.14 to 39.1, respectively). There was no significant difference in histological scores between individuals infected with single-repeat and multiple-repeat Western-type *cagA* (i.e., ABC vs. ABCC or ABCCC). Additionally, there were no significant differences in histological scores between subjects infected with strains containing EPIYA and EPIYT motifs, or between those with identical and non-identical CM motifs (P > 0.05).

Subjects infected with *vacA* s1 strains showed activity and inflammation in the antrum that was significantly greater than in those infected with *vacA* s2 strains (mean [median]; 1.36 [1] vs. 0.75 [1], P = 0.01 and 1.68 [2] vs. 1.17 [1], P = 0.013, respectively). Inflammation and atrophy scores for the antrum were significantly higher in subjects infected with strains containing *vacA* m1 than in those infected with *vacA* m2 strains (mean [median]; 1.70 [2] vs. 1.42 [1], P = 0.05 and 0.90 [1] vs. 0.65 [1], P = 0.047, respectively). In addition, activity in the corpus and OLGA scores were significantly increased in subjects infected with strains containing *vacA* m1, relative to those infected with *vacA* m2 strains (mean [median]; 0.90 [1] vs. 0.58 [1], P = 0.049 and 0.90 [1] vs. 0.65 [1], P = 0.047, respectively). Similarly, subjects who were infected with virulent i and d types (i1 and d1) had inflammation in the antrum and activity in the corpus that was significantly greater than that of subjects infected with *vacA* i2 and d2 strains (P = 0.02 and P = 0.042, respectively). Inflammation and atrophy scores for the antrum, activity in the corpus, and OLGA scores were significantly increased in subjects infected with strains containing *vacA* c1, relative to those infected with *vacA* c2 (for both, P = 0.047).

**Fig 3:**
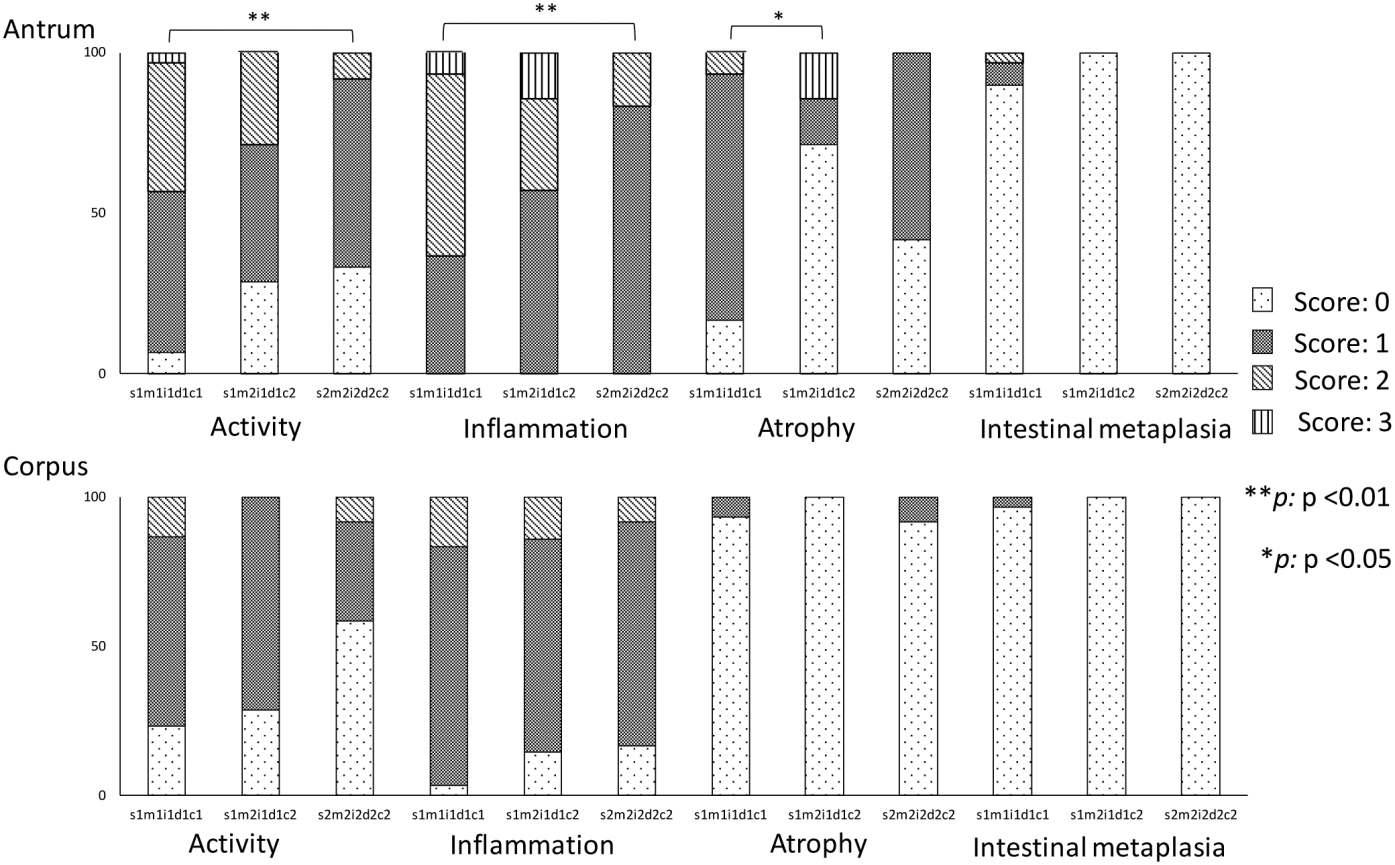
Histological analysis of specimens from subjects infected with combination *vacA* genotypes. Histological severity scores based on the updated Sydney system are indicated on the X-axis and the genotype combinations of *vacA* are described on the Y-axis. In line with the analysis of single sub-genotypes of *vacA*, s1m1i1d1c1 strains induced significantly greater atrophy, activity, and inflammation in the antrum compared to s1m2i1d1c2, s2m2i2d2c2, or s2m2i2d2c2 strains.

Histological analysis of specimens from subjects infected with combination *vacA* genotypes showed that subjects infected with *vacA* s1m1i1d1c1 strains had more antral atrophy than was seen in subjects infected with strains of genotype s1m2i1d1c2 (0.90 [1] vs. 0.57 [0], P = 0.034, Fig 3). Subjects infected with *vacA* s1m1i1d1c1 strains also showed antral activity and inflammation that was increased, relative to activity and inflammation in subjects infected with s2m2i2d2c2 (1.40 [1] vs. 0.75 [1], P = 0.008 and 1.70 [2] vs. 1.17 [1], P = 0.007, respectively). Multivariate analysis showed that subjects infected with *vacA* m1 strains had a significantly higher risk of antral atrophy after adjusting for age, sex, and *cagA* status (OR = 11.90, 95% CI = 1.685 to 83.98), relative to those infected with the m2 genotype.

### Population structure

**Fig 4:**
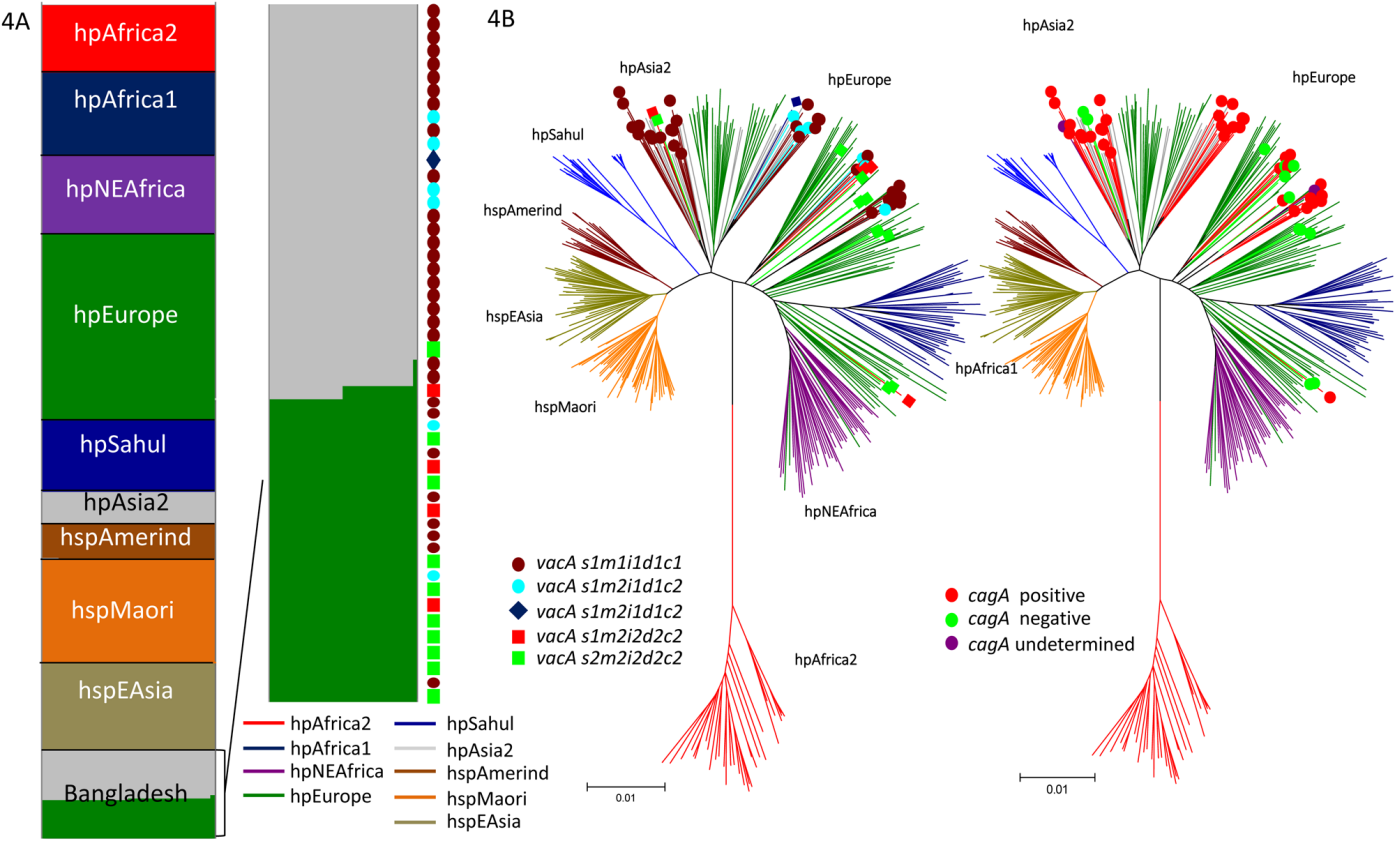
Population analysis of Bangladeshi strains. The number of tentative populations (K) was set from 7 to 15, and 5 to 15 runs were executed for each of the K population components, which are represented in K colors. Each vertical line in the bar chart represents a single strain, and the line colors indicate the population to which each strain might belong. The lengths of the colored lines are proportional to the probability that the strain belongs to that particular population. The population segregated into two populations, hpAsia2 and hpEurope, as confirmed by non-admixture STRUCTURE analysis (4A). We used a different line color to distinguish each population type in both STRUCTURE and in the neighbor-joining tree constructed in 4B (e.g., hpEurope is shown in green, and hpAsia2 is shown in grey). The position of strains in relation to their genotypes are shown in colored boxes or circles.

A total of 51 strains were analyzed using STRUCTURE. Five strains were excluded because one of the seven housekeeping genes was undetectable in the NGS data and because of the poor quality of sequencing results. We investigated the population structure of the Bangladeshi strains using the highest posterior probability of five runs (K = 15). A non-admixture STRUCTURE analysis estimated the geographic origins of each of the ancestral populations. Interestingly, although we obtained samples from subjects of one ethnicity (Bengali), we found that the Bangladeshi strains were divided into two main populations in similar proportions: the hpAsia2 (grey, 54.9%, 28/51) and hpEurope (green, 43.1%, 22/51) populations (Fig 4A). STRUCTURE results were supported by a neighbor-joining tree constructed in MEGA 3.1, using a Kimura-2 parameter model that revealed clear geographic associations for the two populations (Fig 4B). No association between population type and either sex or place of residence (P = 0.07 and P = 0.45) was observed. Histological analysis revealed unexpected results: the subjects infected with hpAsia2 strains showed greater activity and inflammation in the antrum compared to subjects infected with hpEurope strains (1.39 [1.5] vs. 0.95 [1], P = 0.025 and 1.75 [2] vs. 1.36 [1], P = 0.047).

Results of a detailed analysis combining population structure and genotype are shown in Table 4. The hpEurope population contained more *cagA*-negative strains (76.9%, 10/22) compared to the hpAsia2 population (15.4%, 2/28, P = 0.002). In addition, hpAsia2 comprised a higher proportion of the more virulent *vacA* genotype (s1m1i1d1c1) and a lower proportion of the less-virulent genotype (s2m2i2d2c2) compared to hpEurope (75.0%, 21/28 vs. 36.4%, 8/22; 3.6%, 1/28 vs. 40.9%, 9/22, P = 0.001, respectively). Multivariate analysis showed that, compared to subjects infected with *cagA*-negative strains, subjects infected with *cagA*-positive strains, independent of their population types, had a higher risk of antral activity after adjusting for age, sex, and population type, although this difference was not statistically significant (OR = 5.063, 95% CI = 0.819 to 31.304, P = 0.08). Moreover, compared to subjects infected with the s2m2i2d2c2 genotype, those infected with the *vacA* s1m1i1d1c1-only genotype, independent of their population types, had a significantly higher risk of inflammation in the antrum after adjusting for age, sex, and population type (OR = 7.451, 95% CI = 1.027 to 54.077, P = 0.047).

**Table 4:** *H*. *pylori* population and genotypes.

Genotype	Total	*H*. *pylori* population (%)		
		hpAsia2**	hpEurope	hpAsia2-hpEurope
*cagA*-negative	13*	2 (15.4)	10 (76.9)***	0
*cagA*-positive	41**	25 (61.0)	11 (26.8)	1 (2.4)
Group 1: *vacA s1m1i1d1c1*	29	21 (75.0)	8 (36.4) ***	0
Group 2: *vacA s1m2i1d1c2*	6	4 (14.2)	2 (9.1)	0
Group 3: *vacA s1m2i1d1c2*	1	1 (3.6)	0 (0.0)	0
Group 4: *vacA s1m2i2d2c2*	5	1 (3.6)	3 (13.6)	1 (100.0)
Group 5: *vacA s2m2i2d2c2*	10	1 (3.6)	9 (40.9)	0
Total	51	28	22	1

* One strain was excluded because one of the seven housekeeping genes was undetected in the NGS data and because of the poor quality of sequencing results.

**hpAsia2 strains induced greater antral histological severity than hpEurope strains.

*** the hpEurope population contained more *cagA*-negative and less-virulent *vacA* genotypes than the hpAsia2 population.

## Discussion

We confirmed that *H*. *pylori* strains from a Bengali population in Bangladesh were divided into two main populations. We could not confirm which population is older because of our study design; however, we assumed that the hpAsia2 population was distributed evenly in the Indian subcontinent before being reduced through competition with novel strains (hpEurope), as has been seen in previous studies that will be described below. It has been suggested that Ancestral Europe 1 (AE1) originated in Central Asia, where it evolved into hpAsia2, which is commonly found in South Asia (6). A hybridization between AE1 and AE2, which appears to have evolved in northeast Africa, became the more recently diverged hpEurope (4). This supports the surprising results that the *H*. *pylori* of the Iceman—a mummy found in the European Alps—is hpAsia2, with a low proportion of its genome reflecting AE2 ancestry [30], confirming that the introduction of hpAsia2 into the European continent occurred at least 5,000 years ago. However, other studies found that all Indian strains that were sequenced from Native Indian people, who are mainly of Aryan and Dravidian ancestry, have significant homology to the hpEurope population, irrespective of religion (Muslim or Hindu) suggested the possibility that *H*. *pylori* arrived with Indo-Aryan migration (approximately 4,000–10,000 years ago) [31]. In contrast, Ladakhi strains were categorized as mainly hpAsia2, with some hpEurope, irrespective of religion (Muslim or Buddhist) [31], when sequences were compared with sequences of similar strains from a previous publication [32]. The presence of an hpAsia2 population in Bangladeshi strains supports the hypothesis that hpAsia2 was an independent population that was widespread throughout the Indian subcontinent after the Dravidians retreated to avoid Indo-European dominance [33]. This is supported by the difference in the distribution of *cagA* and *vacA* genotypes in the two populations, although their *vacA* m sequences were similar to one another, as well as to strains with the *vacA* m1c allele from India (Fig 1). In fact, our previous report revealed that Nepalese strains also contained an hpAsia2 population, instead of a population specific to Nepal [7]. The hpEurope population spread to Southeast Asia, including Cambodia, Thailand, and Malaysia, likely within the last 3,000 years [34].

Through multivariate analysis, our study also confirmed an unexpected finding that subjects infected with hpAsia2 strains had greater activity and inflammation in the antrum than subjects infected with hpEurope strains. Although no previous study has examined histological severity by *H*. *pylori* population, the hpEurope population is believed to be associated with a higher risk of gastric cancer than is hpAsia2, based on the incidence of gastric cancer in people from each continent (9.4/100,000 vs. 6.7/100,000 for Europe and south-central Asia, respectively; http://globocan.iarc.fr/). This is likely because of the higher proportion of the less-virulent *cagA* and *vacA* genotypes among hpEurope strains that were associated with less severe histological damage. The Bangladeshi strains analyzed in this study had a high proportion of less-virulent *cagA*-positive and *vacA*-positive strains, similar to that observed in strains circulating in neighboring countries (Table 5) [7, 35–40]. Therefore, this is likely only a coincidence and not causal. Previously, we suggested that virulence factors, especially *cagA*, were a better predictor of gastric cancer risk than the ancestral origin of *H*. *pylori* [41]. However, the question remains: why were the less-virulent types of *cagA* and *vacA* not associated with the hpAsia2 population that are typical in South Asia? Another possibility is that antral gastritis predominantly induces a hyperacidity that predisposes an *H*. *pylori*-infected person to gastric metaplasia of the duodenal mucosa, allowing *H*. *pylori* colonization of the duodenum and propagation of duodenal ulceration [8]. South Asia is a region with a high prevalence of duodenal ulcers. Therefore, hpAsia2 strain colonization may be a marker for duodenal ulcer risk in Bangladesh. However, within the hpAsia2 population, only one strain was isolated from a patient with duodenal and gastric ulcers.

**Table 5:** Summary of prevalence and genotypes of *H*. *pylori* in South Asia.

Country	Gastric cancer incidence	Prevalence of *H*. *pylori*	*cagA*- positive	*cagA* type prevalence	*vacA* s1m1
Bangladesh	5.8	47.0%	73.2%	Western-type *cagA* (100.0%)	53.6%
Nepal [7]	12.1	38.4%	100.0%	Western-type *cagA* (94.1%)*	60.8%
India [35–37, 42]	6.1	58.0%	64.0–79.1%	Western-type *cagA* (100.0%)	37.2–59.2%
Pakistan [38]	3.0	61.3%	80.4%	Western-type *cagA* (90.0%)	58.7%
Bhutan [39, 40]	17.2	73.4%	98.6%	East Asian-type *cagA* (91.7%)	33%**

*Includes an East Asian-type and Western-type *cagA* recombinant (23.6%)

**Included in the 38.3% of strains with the s1 m1-m2 chimeric genotype

Our study confirmed that, among Western strains, *vacA* s1m1 and s2m2 strains were exclusively i1 and i2, respectively, and that the s1m2 strains vary in their i types [10]. We also found that the more virulent genotypes (i.e., i1, d1, and c1) were associated with significantly more severe histological scores than were i2, d2, and c2. However, in contrast with results of a previous study in Iran showing that i1- and c1-type strains were strongly associated with gastric adenocarcinoma, irrespective of their *vacA* s or m type or *cag* status [13, 43], we confirmed that in our Bangladesh study, only the *vacA* m region was independently associated with a higher risk of atrophy in the antrum after adjusting for age, sex, and *cagA* status. In addition, all strains containing s1 and m1 possessed i1, d1, and c1, while strains containing s2 and m2 possessed i2, d2, and c2. All strains possessing s1m2i2 also contained d2 and c2. Therefore, in countries such as Bangladesh that have a low prevalence of gastric cancer, the i, d, and c genotypes may simply reflect the s and m regions, and the *vacA* m region may be used as a virulence marker instead of using the overall *vacA* genotype. Moreover, combining the *cagA* and *vacA* genotypes instead of the s region showed that all *cagA*-negative strains possessed m2i2d2c2 and that the m1i1d1c1 genotype was predominant in *cagA*-positive strains, suggesting the possibility that *vacA* genotypes only reflect the presence of these other virulence factors, which primarily represent differences related to human migration [44].

Interestingly, although the first CM motif in half of the strains was different from that of typical Western-type *cagA*-positive strains, the CM motifs consisted of only three types (Table 3). The EPIYA motifs in these strains only consisted of two types (EPIYA and EPIYT), which is a low number in comparison with that found in our previous publication in Okinawa, which identified six types of EPIYA motifs (EPIYA, EPIYT, ESIYA, ESIYT, ELIYA, QPIYA, and EPVYA) [27]. In addition, an analysis of the 300-bp region upstream of the first EPIYA motif showed that no strains contained the deletion that is typically observed in strains from Western countries. Although the same Western-type *cagA* was predominant, our previous report showed that Nepalese strains contained three types of pre-EPIYA sequences (no-deletion type, 6-bp type, 18-bp type) [7]. Moreover, all B-segments of Bangladeshi *cagA* strains contained no chimeric EPIYA segments, and all were categorized as type B_C_, which is typical of Western-type *cagA*-positive strains (Table 5) [1]. These “homogenous” genotypes include a few recombinants among the Bangladeshi strains. In general, recombination is extremely frequent in *H*. *pylori* [45], as a result of adaptation during chronic colonization [46].

All Bangladeshi strains of *H*. *pylori* were categorized as Western-type *cagA*, which is the less-virulent *cagA* genotype [1]. Sequence analysis indicated that the predominant *vacA* genotype of Bangladeshi strains, s1m1, was associated with higher scores related to activity, inflammation, and atrophy when compared to the scores associated with s2m2 *H*. *pylori* strains. However, most of the *vacA* s regions were of the s1a subtype, which is less frequently found in patients with gastric cancer than in those with peptic ulcers and chronic gastritis [47] or is present only in dyspeptic patients [48]. In addition, the predominant m region subtype was m1c, which is specific to South Asian countries with a low ASR of gastric cancer. Moreover, the higher proportion of *vacA* s2 and m2 genotypes, even in *cagA*-positive strains, reflects the low prevalence of gastric cancer-inducing strains, which is in agreement with an *in vitro* study that showed that this genotype fails to induce cell vacuolation [1]. These results might explain, at least in part, the “Asian enigma” in Bangladesh, where the *H*. *pylori* infection rate in the Bangladeshi population is high, but the risk of gastric cancer is low.

There are several limitations in this study. First, the number of strains included was small. Further studies with a larger number of samples, balanced for each diagnosis, will be necessary to better understand the association between virulence factors and clinical outcomes in Bangladesh. Secondly, we obtained samples from a single hospital in Dhaka, which is the capital and largest metropolis in Bangladesh. The physical and cultural landscape varies by region in Bangladesh. Therefore, our results cannot be generalized across all of Bangladesh.

In conclusion, we revealed that Bangladeshi strains segregated into two main populations with different genotypes. The low incidence of gastric cancer in Bangladesh might be attributable to the high proportion of less -virulent genotypes, which may be a better predictor of gastric cancer risk than is the ancestral origin of *H*. *pylori* strains. Finally, we determined that the *vacA* m region, instead of the overall *vacA* genotype, can be used as a virulence marker.
